# Multi-strategy integrated Gorilla Troops Optimizer for solving global optimization and engineering design problems

**DOI:** 10.1038/s41598-025-19427-3

**Published:** 2025-10-09

**Authors:** Zhijun Teng, Liangcen Gu, Mingyang Sun, Mugang He

**Affiliations:** 1 School of Electrical Engineering, Northeast Electric Power University, Jilin, 132000 China; 2 China Telecom Corporation Limited Jilin Branch, Changchun, 130000 China

**Keywords:** Gorilla troops optimizer, Latin hypercube sampling, Levy flight, Cauchy inverse cumulative distribution, Engineering problems, Mathematics and computing, Applied mathematics, Computational science

## Abstract

Inspired by the intricate group dynamics of wild gorilla populations, the Artificial Gorilla Troops Optimizer (GTO) represents a novel approach in swarm intelligence. Despite its effectiveness in performing global exploration, GTO is prone to early convergence and can easily become stuck in local optima, especially when addressing optimization problems with intricate constraints and rugged search spaces. To overcome these limitations, this paper introduces the Multi-Strategy Integrated Gorilla Troops Optimizer (MSIGTO), which integrates Latin Hypercube Sampling (LHS), Lévy Flight (LF), and the Cauchy Inverse Cumulative Distribution Operator (CICDO). The diversity of the initial population is enhanced through LHS, and the exploration and convergence characteristics of the algorithm are further improved by LF and CICDO. To validate its effectiveness, MSIGTO is compared with 8 representative population-based optimization algorithms. Experimental evaluations on the 2017 IEEE Congress on Evolutionary Computation (CEC2017) and 2022 IEEE Congress on Evolutionary Computation (CEC2022) benchmark suites demonstrate that MSIGTO achieves a Friedman mean rank of 1.48 on 100 dimensional problems and 1.75 on 20 dimensional problems, respectively. These results indicate superior global exploration capability, convergence efficiency, and solution robustness compared with 8 population-based optimization algorithms. The algorithm’s practicality was further verified on four constrained real-world engineering problems, including the speed reducer design problem, the gear train design problem, the multiple disk clutch brake design problem, and the selective harmonic elimination pulse-width modulation problem for three-level inverters. Overall, the results confirm that MSIGTO is an effective optimizer with broad potential for engineering optimization applications.

## Introduction

The objective of optimization problems is to identify optimal decision variables from a finite set to maximize or minimize a given objective function^1^. In general, optimization methods fall into two main types: deterministic and stochastic. Conventional methods relying on gradient information, including Newton-based strategies^2^ and conjugate direction techniques^3^, often encounter difficulties as the problem dimensionality increases, particularly under complex constraints and non-differentiable black-box settings. In contrast, stochastic methods, particularly metaheuristic algorithms, offer self-organizing and self-learning capabilities without relying on gradient information. These algorithms are known for their high computational efficiency and low cost^4^, making them widely applicable to real-world optimization tasks^5^. Metaheuristic approaches are typically sorted into four primary groups: evolutionary algorithms, exemplified by Genetic Algorithms (GA)^6^ and Linear-SHADE (LSHADE)^7^; nature-inspired algorithms, including the Tornado Optimizer with Coriolis Force (TOC)^8^, Fata Morgana Algorithm (FATA)^9^ and the Candle Flame Optimization (CFO)^10^; human behavior-based algorithms, such as the Alpine Skiing Optimization (ASO)^11^, Dream Optimization Algorithm (DOA)^12^, Negotiators Algorithm (NA)^13^, Farmer and Seasons Algorithm (FSA)^14^, Singer Optimization Algorithm (SOA)^15^, Social Rumor Propagation Optimization (SRPO)^16^, Motorbike Courier Optimization (MCO)^17^, Perfumer Optimization Algorithm (POA)^18^, Makeup Artist Optimization Algorithm (MAOA)^19^, and the Builder Optimization Algorithm (BOA)^20^; swarm intelligence algorithms, including the Artificial Gorilla Troops Optimizer (GTO)^21^, Hunger Games Search (HGS)^22^, African Vultures Optimization Algorithm (AVOA)^23^, Pelican Optimization Algorithm (POA)^24^, Parrot Optimizer (PO)^25^, Rabbit and Turtle Algorithm (RTS)^26^, Gyro Fireworks Algorithm (GFA)^27^, Frigate Bird Optimizer (FBO)^28^, and the Fishing Cat Optimizer (FCO)^29^.

Given the No Free Lunch (NFL) theorem, which asserts that no single optimizer is superior for all scenarios^30^, researchers have been inspired to continually improve algorithmic frameworks^31^. In 2021, Abdollahzadeh et al. presented the GTO algorithm^21^, which emulates the group coordination and dominance structure typical of silverback gorilla communities. It has demonstrated strong effectiveness across a range of applications, including photovoltaic parameter estimation^32^, medical feature selection^33^, fuel cell parameter extraction^34^, fractional-order PID controller tuning^35^, reservoir operation^36^, and power system stabilizer optimization^37^. Building upon the original GTO algorithm, numerous variants have been proposed by researchers to enhance its optimization performance: Chaotic Gorilla Troops Optimizer (CGTO) with chaotic strategies^38^, Artificial Differential Evolution Gorilla Troops Optimizer (ADEGTO) integrating Differential Evolution^39^, Hybrid algorithm-based AOA and GTO (HAOAGTO) combining Arithmetic Optimization^40^, Opposition-based learning and Parallel strategies Artificial Gorilla Troop Optimizer (OPGTO) using opposition-based learning and parallel strategies^41^, and Quantum Artificial Gorilla Troops Optimizer (QGTO) applying quantum computing concepts^42^. Table 1 summarizes the representative swarm intelligence algorithms, highlighting their main contributions, strengths, and limitations to clarify the research gap addressed by our proposed MSIGTO.

**Table 1:** Summary of representative swarm intelligence algorithms and their characteristics.

Algorithm	Year	Main contributions	Strengths	Limitations
Linear-SHADE (LSHADE)	2014	Adaptive differential evolution with linear population size reduction	Excellent convergence speed; strong performance on CEC benchmarks	May require fine- tuning; less effective on highly constrained problems
Gorilla Troops Optimizer (GTO)	2021	Swarm optimizer inspired by gorilla social behavior	Balanced exploration and exploitation; competitive on multimodal functions	Tends to early convergence; diversity loss in later stages
African Vultures Optimization Algorithm (AVOA)	2021	Mimics African vultures’ foraging strategies	Strong exploration; simple implementation	Susceptible to slow convergence on unimodal problems
Hunger Games Search (HGS)	2021	Based on hunger- driven search dynamics	Good global search ability; suiTable for diverse landscapes	May oscillate in convergence; parameter sensitivity
Pelican Optimization Algorithm (POA)	2022	Inspired by pelican hunting and diving behavior	Efficient search transitions; low computational cost	Weak in fine-tuning near optimum
Weighted Mean of Vectors (INFO)	2022	Combines weighted mean of vectors for population updates	Good balance of global and local search	Performance degrades on highly rugged landscapes
Parrot Optimizer (PO)	2024	Parrot communication- inspired optimizer	Strong in information sharing and convergence	Limited benchmark validation; scalability uncertain
Fata Morgana Algorithm (FATA)	2024	Fata Morgana mirage phenomenon- based optimizer	Innovative exploration mechanism; good escape from local minima	Immature parameter control; may require hybridization
Gyro Fireworks Algorithm (GFA)	2024	Gyro fireworks explosion-based search strategy	Strong diversification; flexible parameter control	Computationally expensive for large- scale problems
Frigate Bird Optimizer (FBO)	2024	Frigate bird migration and hunting patterns	Good balance of exploration and exploitation	Lack of theoretical analysis; moderate convergence speed
Fishing Cat Optimizer (FCO)	2025	Inspired by fishing cat hunting tactics	Effective handling of multimodal landscapes	Limited tests on constrained problems
Makeup Artist Optimization Algorithm (MAOA)	2025	Makeup artist skill metaphor for solution refinement	Good local exploitation ability	May get trapped in local optima; weak global exploration
Candle Flame Optimization (CFO)	2025	Candle flame flame-flickering behavior for search	Simple implementation; fast convergence in low dimensions	Performance drops in high-dimensional or noisy problems

Although the GTO algorithm demonstrates promising performance, it still suffers from several limitations, including insufficient population diversity during initialization, limited capability to escape from local optima, and inadequate stability. To address these issues, this study proposes several novel strategies aimed at enhancing the performance of the GTO algorithm and overcoming its inherent drawbacks. The major improvements are summarized as follows:

1. Latin Hypercube Sampling (LHS): The LHS strategy^43^ is employed to enhance the diversity of the population during initialization, thereby providing a more comprehensive exploration of the search space at the early stage of the algorithm.
2. Lévy Flight (LF): The LF strategy^44^ is incorporated to improve the algorithm’s ability to escape from local optima and to strengthen its search performance during the exploitation phase.
3. Cauchy Inverse Cumulative Distribution Operator (CICDO): The CICDO^45^ is utilized to enhance the stability of the algorithm, which in turn accelerates its convergence toward high-quality solutions.

To evaluate MSIGTO, the CEC2017 benchmark suite^46^ and CEC2022 benchmark suite^47^ are utilized, and a performance comparison is carried out with seven competing algorithms—Parrot Optimizer (PO), Fata Morgana Algorithm (FATA), Pelican Optimization Algorithm (POA), Weighted Mean of Vectors (INFO)^34^, Hunger Games Search (HGS), Linear-SHADE (LSHADE), African Vultures Optimization Algorithm (AVOA)—as well as the original GTO. Furthermore, the algorithm is additionally validated in terms of stability and effectiveness by applying it to four practical constrained engineering problems.

The structure of this paper is as follows: section “The proposed optimization algorithm” offers an introduction to the original Gorilla Troops Optimizer (GTO) and presents the proposed improvements incorporated in the Multi-Strategy Integrated GTO (MSIGTO). Section “Experimental results and discussion” evaluates the algorithm’s performance using the 2017 IEEE Congress on Evolutionary Computation (CEC2017) and 2022 IEEE Congress on Evolutionary Computation (CEC2022) benchmark suites. In section “Engineering design problems”, the application of MSIGTO to four real-world engineering design problems is explored. Section “Discussion” provides a comprehensive discussion of the experimental results, analyzing the algorithm’s strengths, limitations, and potential areas for further enhancement. Finally, section “Conclusion” provides a summary of the findings and suggests potential directions for future research.

## The proposed optimization algorithm

### Original algorithm GTO

Originally introduced in 2021, the Artificial Gorilla Troops Optimizer (GTO) represents a nature-inspired optimization technique modeled on the collective behaviors and hierarchical interactions observed in wild gorilla groups^21^. Within each troop, a dominant adult male, known as the “silverback,” assumes leadership. In GTO, five operators are designed to mimic the collective social dynamics observed in gorilla troops. The exploration phase in GTO incorporates three strategies: migrating to an unknown area, migrating to a known area, and moving toward other gorillas. Meanwhile, the exploitation phase involves two strategies: following the silverback and competing for adult female gorillas.

During the exploration phase, each individual in the population corresponds to a potential solution. At every iteration, the individual with the optimal fitness is designated as the ‘silverback. *r*_*1*_, *r*_*2*_, *r*_*3*_, *rand* and *p* in (1) are uniformly sampled from the [0,1] range. The parameter *p* specifies the likelihood of transitioning toward an unknown region. *X(t)* shows the present position of a gorilla, whereas *GX(t + 1)* signifies its updated position in the subsequent iteration. *b*_*up*_ defines the upper bound, while *b*_*low*_ defines the lower limit of the search space. *X*_*r*_*(t)* refers to a randomly selected individual from the current population, whereas *GX*_*r*_*(t)* represents one chosen at random from the candidate pool. *C*, *L*, and *H* are computed using (2), (4), and (5), in that order.

1 $$GX(t+1)=\left\{ {\begin{array}{*{20}{c}} {({b_{up}} - {b_{low}}) \times {r_1}+{b_{low}}\;\;\;\;\;\;\;\;\;\;\;\;\;\;\;\;\;\;\;\;rand<p,} \\ {({r_2} - C) \times {X_r}(t)+L \times H\;\;\;\;\;\;\;\;\;\;\;\;\;\;\;rand \geqslant 0.5,} \\ {X(t) - L \times (L \times (X(t) - G{X_r}(t))\;\;\;\;\;\;\;\;\;\;\;\;\;\;\;\;\;\;} \\ {\;\;\;\;\;\;\;+{r_3} \times (X(t) - G{X_r}(t)))\;\;\;\;\;\;\;\;rand<0.5,} \end{array}} \right.$$

2 $$C=F \times \left( {1 - \frac{{Itr}}{{MaxItr}}} \right)$$

3 $$F=\cos (2 \times {r_4})+1$$

4 $$L=C \times l$$

5 $$H=Z \times X(t)$$

Equation (2) defines *Itr* as the current iteration index, with *MaxItr* indicating the upper limit of iterations. The parameter *F*, as defined in (3), incorporates the cosine function and a random variable *r*_*4*_, uniformly drawn from the interval [0,1]. Parameter *L* models the leadership ability of the silverback, calculated using (4), with *l* being a stochastic variable that lies within the interval [0,1]. The variable *Z* is uniformly distributed within the bounds of [− *C*, *C*].

The exploitation phase is broken down into two main strategies: following the silverback and competing for adult females. The choice between them depends on the value of *C* from (2) compared to a predefined threshold *W*. When *C ≥ W*, the algorithm follows the behavior outlined in (6), in which *X(t)* refers to the current location of a gorilla and *X*_*silverback*_ corresponds to the silverback’s position. Equation (4) defines parameter *L*, and (7) provides the formulation for *M*. *GX*_*i*_*(t)* indicates the current coordinate of the i-th gorilla at iteration *t*. *N* is the total population size, and *g* is computed according to (8).

6 $$GX(t+1)=L \times M \times (X(t) - {X_{silverback}})+X(t)$$

7 $$M = \left( {\left| {\frac{1}{N}\mathop \sum \limits_{{i = 1}}^{N} GX_{i} (t)} \right|^{g} } \right)^{{\frac{1}{g}}}$$

8 $$g={2^L}$$

When *C < W*, the competition strategy is triggered, as defined in (9). *GX*_*i*_*(t)* indicates the location of a gorilla in the search space at iteration *t*. *rand* and *r*_*5*_ are uniformly distributed random variables within the interval [0,1].

Both *rand* and *r*_*5*_ are random variables that follow a uniform distribution over the range [0,1]. The parameter *Q*, representing impact force, is computed using (10), while *A* denotes the intensity of conflict and *β* is a predefined coefficient. According to (12), *E* is drawn from a normal distribution conditioned on the problem dimension when *rand ≥ 0.5*; in contrast, for *rand < 0.5*, *E* is sampled from the identical distribution utilizing a different seed.

9 $$G{X_i}({\text{t}})={X_{silverback}} - ({X_{silverback}} \times Q - X(t) \times Q) \times A$$

10 $$Q=2 \times {r_5} - 1$$

11 $$A=\beta \times E$$

12 $$E=\left\{ {\begin{array}{*{20}{c}} {{N_1},\;\;\;\;\;rand \geqslant 0.5} \\ {{N_2},\;\;\;\,\;rand<0.5} \end{array}} \right.$$

### Proposed MSIGTO algorithm

Like many other metaheuristic approaches, GTO is prone to challenges such as reduced population diversity and premature convergence to suboptimal solutions. To overcome these issues, the present study proposes an improved version of the algorithm, MSIGTO. The core concepts of MSIGTO are outlined as follows: (1) Utilizing the LHS method to improve the initial population’s spread and representation across the search space. (2) Integrating CICDO and LF strategies to refine the two exploitation phases in GTO, with the goal of strengthening local search performance and ensuring greater consistency and efficiency in approaching the optimal solution. The improvements are further discussed in detail in the next sections.

1. Latin hypercube sampling.

Research findings suggest that proper initialization is vital to enhancing both convergence efficiency and solution quality in optimization algorithms^49^. LHS represents a structured probabilistic sampling approach applied to explore multidimensional parameter spaces^50^. LHS efficiently covers the entire parameter space with fewer samples by ensuring that each variable is distributed across a fully stratified feature space using auxiliary variables^51^.

2. Levy Flight.

Lévy Flight (LF) is a stochastic process that captures the dynamics of systems exhibiting infrequent but extensive displacements^44^. As an effective strategy for randomly searching an unknown space for optimal solutions, LF is widely used in both natural and artificial systems. It has been incorporated into the development and improvement of various metaheuristic algorithms, including AVOA optimization, DLF-ChoA optimization^52^. Equation (13) presents the step size formula, in which *u* and *v* are sampled from a normal distribution, and *λ* is a fixed parameter within the interval [0, 2].

13 $$LF(x)=0.01 \times \frac{{u \times \sigma }}{{|\nu {|^{\frac{1}{\lambda }}}}}\;\;\;$$

14 $$\sigma ={\left( {\frac{{{\text{\varvec{\Gamma}}}(1+\lambda ) \times \sin (\frac{{\pi \lambda }}{2})}}{{{\text{\varvec{\Gamma}}}(\frac{{1+\lambda }}{2}) \times \lambda \times {2^{(\frac{{\lambda - 1}}{2})}}}}} \right)^{\frac{1}{\lambda }}}$$

During the GTO algorithm’s exploitation phase, the LF strategy is integrated into the “Following the Silverback” mechanism. Specifically, when C ≥ W, the LF operator is added to Eq. (6). The improved version is formulated in Eq. (15). The LF operator provides a more profound search pattern, enhancing the efficiency of global exploration.

15 $$GX(t+1)=L \times M \times (X(t) - {X_{silverback}}) \oplus LF(x)+X(t)$$

*L* and *M* are obtained from (4) and (7) of the original GTO algorithm, respectively. The symbol ⊕ denotes element-wise multiplication.

3.Cauchy Inverse Cumulative Distribution Operator.

Equation (16) defines the probability distribution function of the CICD, where the location parameter *m* corresponds to the peak’s location, and the scale parameter *n* determines the half-width at the maximum. The cumulative distribution function (CDF) is given in (17).

16 $$f(x;m,n)=\frac{1}{{\pi n\left[ {1+{{\left( {\frac{{x - m}}{\gamma }} \right)}^2}} \right]}}$$

17 $$F(x;m,n)=\frac{1}{\pi }\arctan \left( {\frac{{x - m}}{n}} \right)+\frac{1}{2}$$

Given that the cumulative distribution function (CDF) of the Cauchy distribution serves as its inverse for computational purposes, the inverse transformation technique is employed to generate random values that adhere to a uniform distribution. The formula for the inverse cumulative distribution function (Inverse CDF) of the Cauchy distribution is provided in (18).

18 $$x={F^{ - 1}}(u;m,n)=m+n \cdot \tan \left[ {\pi \cdot \left( {\eta - \frac{1}{2}} \right)} \right]$$

19 $$\eta ={\text{randn}}(1,d)$$

During the exploitation phase of the GTO algorithm, the “Competition for adult females” mechanism is enhanced by integrating the Cauchy Inverse Cumulative Distribution Operator (CICDO), which is applied when *C < W*. The modified equation is presented in (20). This improvement shortens the gap between individual gorillas and the silverback, resulting in a faster decrease in the final step size and a more rapid approach to the optimal solution.

20 $$G{X_i}({\text{t}})={X_{silverback}} - ({X_{silverback}} \times Q - X(t) \times Q) \times \tan \left[ {\pi \cdot \left( {\eta - \frac{1}{2}} \right)} \right]$$

**Fig. 1:**
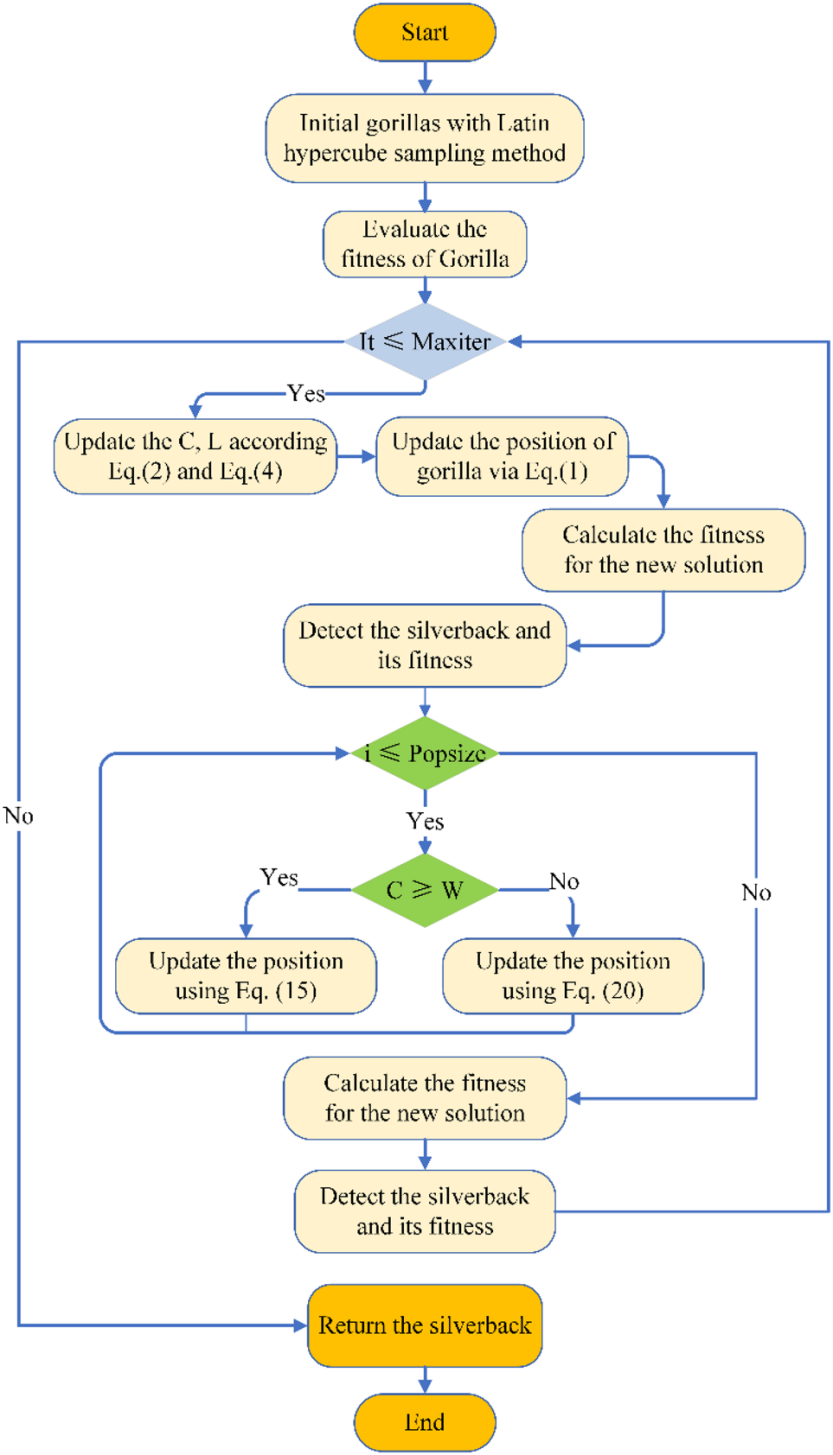
The flowchart of the MSIGTO method.

The parameter *Q* is computed using Eq. (10) to simulate impact force, while *η* is determined by Eq. (19). Figure 1 illustrates the flowchart of the MSIGTO algorithm.

### Computational complexity analysis

The computational complexity of an algorithm is an essential measure for evaluating its effectiveness in solving optimization tasks. For metaheuristic algorithms, key factors such as the population size (N), problem dimension (D), and the maximum iteration count (T) play significant roles in determining the overall complexity. The time complexity of the GTO algorithm can be expressed as O(2*TND* + *TN* + *N*). Since the dimension *D* in the LHS initialization method is treated as constant, the initialization phase of MSIGTO has a time complexity of O(*N*). As demonstrated through mathematical proofs in^38^, the incorporation of the LF strategy does not contribute to an increase in the time complexity of optimization algorithms, meaning its incorporation in the exploitation phase as a multiplicative factor imposes no additional computational cost. Additionally, in MSIGTO, the CICDO replaces the original algorithm’s step-size correction factor. Both components have a time complexity of O(*D*), so this modification does not alter the overall complexity. Therefore, the exploitation phase retains the original time complexity, and the time complexity of MSIGTO remains O(2*TND* + *TN* + *N*).

## Experimental results and discussion

The effectiveness of the proposed MSIGTO algorithm is evaluated using the CEC2022 benchmark suite, which includes unimodal, basic, hybrid, and composition functions. These functions are treated as black-box optimization problems^47^. To ensure a comprehensive comparison, various statistical metrics, including mean, standard deviation (STD), and p-values obtained from the Wilcoxon rank-sum test, are employed. Apart from the original GTO, MSIGTO is assessed alongside seven leading algorithms: PO, FATA, INFO, Pelican Optimization Algorithm (POA) HGS, AVOA, and LSHADE. Each algorithm is performed under consistent experimental conditions.

The testing environment consists of a system with an Intel Core i5-13400 F CPU, 32 GB of RAM, running Windows 10 (64-bit), and utilizing MATLAB R2023a for simulations. The parameter settings for the experiments are provided in Table 2.

**Table 2:** Parameters setting.

Algorithms	Parameters setting
GTO	β = 3, W = 0.8, *P* = 0.03
MSIGTO	W = 0.8, *P* = 0.03
PO	No such parameter
FATA	arf = 0.2
INFO	No such parameter
POA	No such parameter
HGS	VC2 = 0.03
AVOA	p1 = 0.6, p2 = 0.4, p3 = 0.6 L1 = 0.8, L2 = 0.2, w = 2.5
LSHADE	*p* = 0.11, arc_rate = 1.4, H = 5

### Results and analysis of the CEC2017 benchmark functions

The experiments are conducted with a maximum of 500 iterations, a population size of 30, and a problem dimensionality of 100. Each algorithm is executed independently 30 times. The CEC2017 benchmark suite consists of 29 test functions (Note that the F2 function has been removed from the benchmark set), which include Unimodal Functions (F1, F3), Simple Multimodal Functions(F4–F10), Hybrid Functions (F11–F20), and Composition Functions (F21–F30)^46^. The experimental results, which encompass the mean, standard deviation, and p-values, are presented in Table 3. Based on mean values, MSIGTO achieves the best performance on 20 of these functions (except F3, F6, F9, F14, F18, F23, F24, F26, F27, F28). With respect to standard deviation, MSIGTO attained the best results on 15 of the 29 functions, indicating that the algorithm exhibits favorable stability. The statistical significance of the results was examined using the Wilcoxon rank-sum test at a 5% significance level. This non-parametric approach was adopted to verify whether the observed performance differences between two algorithms were statistically meaningful. A threshold of 0.05 for the p-value was applied, where values above this threshold indicate a lack of statistical significance and are shown in bold in Table 3. Overall, MSIGTO achieved statistically significant improvements compared with most competing algorithms, except for GTO on F9 and for AVOA and HGS on F4, F6, and F12.

**Table 3:** Performance comparison results of function optimization on CEC2017.

Function	Index	MSIGTO	GTO	PO	FATA	INFO	POA	HGS	AVOA	LSHADE
F1	Mean	**1.98E + 09**	2.09E + 10	8.53E + 10	9.90E + 10	1.62E + 10	1.53E + 11	1.19E + 10	3.58E + 10	5.36E + 10
	STD	**8.87E + 08**	1.23E + 10	1.33E + 10	1.39E + 10	7.26E + 09	1.80E + 10	3.04E + 09	8.93E + 09	1.07E + 11
	p-value	null	3.26E-09	1.11E-52	4.07E-78	3.53E-02	1.24E-94	1.51E-01	3.07E-22	2.54E-11
F3	Mean	3.11E + 05	**2.49E + 05**	3.80E + 05	3.29E + 05	3.94E + 05	3.08E + 05	5.66E + 05	4.05E + 05	1.35E + 06
	STD	**1.65E + 04**	2.55E + 04	3.76E + 04	3.60E + 04	7.95E + 04	3.14E + 04	1.92E + 05	1.32E + 05	4.78E + 05
	p-value	null	1.82E-48	3.07E-143	5.77E-148	4.11E-152	2.86E-29	2.54E-153	7.09E-142	7.21E-158
F4	Mean	**1.29E + 03**	2.44E + 03	1.16E + 04	1.82E + 04	2.15E + 03	2.71E + 04	1.82E + 03	4.41E + 03	1.50E + 04
	STD	**1.46E + 02**	5.83E + 02	2.12E + 03	7.41E + 03	4.58E + 02	7.45E + 03	3.93E + 02	1.22E + 03	3.21E + 04
	p-value	null	2.81E-08	7.15E-29	1.05E-85	2.53E-06	1.71E-88	**1.68E-01**	1.78E-08	2.31E-89
F5	Mean	**1.26E + 03**	1.38E + 03	1.79E + 03	1.73E + 03	1.31E + 03	1.61E + 03	1.39E + 03	1.47E + 03	1.65E + 03
	STD	7.12E + 01	5.21E + 01	6.31E + 01	**3.73E + 01**	7.09E + 01	5.00E + 01	8.69E + 01	5.88E + 01	2.80E + 02
	p-value	null	6.44E-32	4.52E-102	9.04E-121	1.63E-15	4.99E-63	6.68E-31	1.36E-50	1.75E-114
F6	Mean	6.51E + 02	6.68E + 02	6.95E + 02	6.85E + 02	6.60E + 02	6.81E + 02	6.51E + 02	6.69E + 02	**6.30E + 02**
	STD	4.21E + 00	4.19E + 00	5.93E + 00	4.74E + 00	5.38E + 00	**3.21E + 00**	4.82E + 00	3.70E + 00	2.86E + 01
	p-value	null	2.18E-32	7.06E-112	5.11E-108	4.10E-07	3.53E-44	**1.01E-01**	1.92E-33	8.89E-16
F7	Mean	**2.49E + 03**	3.12E + 03	3.62E + 03	3.43E + 03	2.89E + 03	3.50E + 03	2.72E + 03	3.24E + 03	2.51E + 03
	STD	1.91E + 02	1.59E + 02	1.39E + 02	1.44E + 02	2.08E + 02	**9.33E + 01**	2.11E + 02	1.35E + 02	1.13E + 03
	p-value	null	5.78E-72	7.78E-90	1.75E-49	3.90E-05	8.89E-102	1.65E-05	7.99E-55	2.18E-03
F8	Mean	**1.63E + 03**	1.81E + 03	2.27E + 03	2.19E + 03	1.69E + 03	2.07E + 03	1.78E + 03	1.91E + 03	1.93E + 03
	STD	9.71E + 01	8.43E + 01	7.88E + 01	**6.51E + 01**	1.07E + 02	6.53E + 01	1.00E + 02	6.60E + 01	2.49E + 02
	p-value	null	1.64E-13	4.37E-68	1.17E-49	4.04E-17	1.09E-27	4.02E-40	9.74E-07	7.45E-13
F9	Mean	2.72E + 04	2.80E + 04	6.39E + 04	5.86E + 04	**2.64E + 04**	4.18E + 04	3.50E + 04	3.25E + 04	6.79E + 04
	STD	**2.00E + 03**	2.45E + 03	6.93E + 03	7.51E + 03	2.92E + 03	3.46E + 03	5.51E + 03	3.04E + 03	5.11E + 04
	p-value	null	**4.33E-01**	7.83E-58	8.75E-49	3.25E-36	1.54E-24	4.31E-23	**4.09E-01**	1.13E-23
F10	Mean	**1.56E + 04**	1.98E + 04	2.76E + 04	3.21E + 04	1.82E + 04	2.07E + 04	1.87E + 04	1.92E + 04	3.28E + 04
	STD	1.42E + 03	3.59E + 03	1.97E + 03	**7.71E + 02**	1.57E + 03	1.17E + 03	1.30E + 03	1.83E + 03	1.62E + 03
	p-value	null	**5.55E-01**	4.52E-91	4.49E-158	3.21E-11	5.99E-10	7.29E-11	3.99E-02	4.61E-178
F11	Mean	**2.98E + 04**	3.13E + 04	1.66E + 05	9.66E + 04	3.02E + 04	8.32E + 04	6.33E + 04	1.36E + 05	3.67E + 05
	STD	**6.37E + 03**	9.25E + 03	2.60E + 04	1.60E + 04	9.48E + 03	1.64E + 04	2.69E + 04	3.45E + 04	1.90E + 05
	p-value	null	9.68E-16	2.48E-107	5.92E-04	8.11E-01	2.38E-06	8.41E-19	1.09E-99	3.49E-173
F12	Mean	**1.66E + 08**	8.26E + 08	2.06E + 10	3.01E + 10	6.09E + 08	6.80E + 10	1.41E + 09	3.58E + 09	1.52E + 10
	STD	**6.77E + 07**	4.73E + 08	6.44E + 09	8.12E + 09	6.76E + 08	1.68E + 10	1.31E + 09	2.00E + 09	1.94E + 10
	p-value	null	1.73E-07	2.56E-47	2.15E-82	7.67E-08	9.57E-77	**3.93E-01**	2.00E-12	9.14E-90
F13	Mean	**2.68E + 04**	1.47E + 05	2.27E + 09	2.41E + 09	1.17E + 06	1.61E + 10	1.67E + 07	5.39E + 07	1.59E + 08
	STD	**1.10E + 04**	1.50E + 05	1.23E + 09	9.01E + 08	5.51E + 06	6.70E + 09	4.21E + 07	1.92E + 08	4.79E + 08
	p-value	null	1.90E-24	8.96E-103	2.38E-113	2.70E-02	1.32E-139	1.29E-10	2.31E-66	2.39E-79
F14	Mean	1.58E + 06	**1.20E + 06**	2.00E + 07	8.36E + 06	1.33E + 06	6.65E + 06	7.71E + 06	1.22E + 07	6.57E + 07
	STD	**5.78E + 05**	6.63E + 05	6.68E + 06	4.49E + 06	6.93E + 05	3.04E + 06	4.31E + 06	5.53E + 06	5.01E + 07
	p-value	null	4.18E-37	9.77E-75	4.29E-65	4.29E-13	5.75E-55	1.46E-70	1.06E-91	9.42E-136
F15	Mean	**5.43E + 03**	2.75E + 04	3.14E + 08	1.48E + 08	1.69E + 04	5.17E + 09	1.01E + 06	1.24E + 06	1.14E + 07
	STD	**2.63E + 03**	1.16E + 04	2.12E + 08	1.13E + 08	1.05E + 04	3.07E + 09	2.56E + 06	4.72E + 06	3.38E + 07
	p-value	null	1.02E-03	2.83E-61	7.25E-62	1.21E-04	1.34E-73	3.92E-07	7.54E-12	5.49E-26
F16	Mean	**5.68E + 03**	6.89E + 03	1.29E + 04	1.02E + 04	6.51E + 03	1.20E + 04	7.05E + 03	8.59E + 03	1.19E + 04
	STD	**7.49E + 02**	8.55E + 02	1.60E + 03	9.84E + 02	7.95E + 02	1.69E + 03	7.78E + 02	1.04E + 03	1.14E + 03
	p-value	null	9.02E-09	8.33E-109	2.01E-95	1.10E-03	7.39E-57	1.69E-02	1.06E-12	6.41E-90
F17	Mean	**5.15E + 03**	6.28E + 03	1.41E + 04	7.16E + 04	5.88E + 03	7.54E + 04	5.68E + 03	6.48E + 03	9.48E + 03
	STD	**5.43E + 02**	6.96E + 02	1.27E + 04	1.64E + 05	6.26E + 02	1.34E + 05	5.98E + 02	7.18E + 02	1.25E + 03
	p-value	null	4.84E-28	3.75E-91	2.67E-109	7.42E-20	3.29E-73	1.36E-24	4.10E-47	5.73E-67
F18	Mean	2.52E + 06	**2.15E + 06**	2.14E + 07	2.00E + 07	2.19E + 06	7.69E + 06	1.68E + 07	7.75E + 06	1.11E + 08
	STD	**8.13E + 05**	1.05E + 06	8.83E + 06	8.99E + 06	1.08E + 06	5.62E + 06	9.35E + 06	4.10E + 06	8.78E + 07
	p-value	null	9.27E-09	8.74E-57	2.14E-51	1.64E-75	4.21E-01	3.67E-115	3.69E-02	1.14E-131
F19	Mean	**6.09E + 03**	1.57E + 05	2.76E + 08	2.08E + 08	3.66E + 04	4.28E + 09	2.15E + 05	6.13E + 06	2.18E + 07
	STD	**4.01E + 03**	3.91E + 05	1.75E + 08	1.60E + 08	5.19E + 04	3.71E + 09	3.30E + 05	5.48E + 06	9.92E + 07
	p-value	null	2.27E-26	2.36E-66	1.30E-96	5.95E-01	3.79E-131	5.21E-06	2.49E-51	1.98E-17
F20	Mean	**4.89E + 03**	5.39E + 03	6.56E + 03	7.53E + 03	5.57E + 03	5.46E + 03	5.53E + 03	6.00E + 03	8.22E + 03
	STD	3.82E + 02	5.19E + 02	5.38E + 02	**3.55E + 02**	5.32E + 02	4.08E + 02	5.55E + 02	5.07E + 02	4.61E + 02
	p-value	null	1.47E-52	3.74E-11	3.95E-85	3.59E-01	8.90E-01	3.07E-16	5.89E-44	7.26E-146
F21	Mean	**3.05E + 03**	3.46E + 03	4.12E + 03	3.94E + 03	3.30E + 03	3.94E + 03	3.22E + 03	3.70E + 03	3.43E + 03
	STD	9.67E + 01	1.51E + 02	1.73E + 02	1.04E + 02	1.74E + 02	1.59E + 02	**9.50E + 01**	1.91E + 02	2.92E + 02
	p-value	null	6.26E-27	5.09E-107	2.27E-122	1.49E-37	1.34E-114	1.80E-14	1.09E-106	1.21E-03
F22	Mean	**1.99E + 04**	2.45E + 04	3.05E + 04	3.38E + 04	2.18E + 04	2.45E + 04	2.12E + 04	2.22E + 04	3.44E + 04
	STD	1.31E + 03	4.27E + 03	1.48E + 03	**6.48E + 02**	1.71E + 03	1.39E + 03	1.40E + 03	2.04E + 03	1.62E + 03
	p-value	null	7.89E-13	4.63E-67	8.72E-119	5.05E-05	2.36E-19	2.50E-03	9.32E-01	3.89E-156
F23	Mean	3.56E + 03	4.18E + 03	5.14E + 03	4.74E + 03	3.92E + 03	5.05E + 03	**3.52E + 03**	4.36E + 03	3.95E + 03
	STD	1.58E + 02	2.61E + 02	2.18E + 02	2.26E + 02	1.77E + 02	2.70E + 02	**8.07E + 01**	2.14E + 02	3.33E + 02
	p-value	null	2.33E-25	7.14E-135	3.82E-144	9.04E-06	5.14E-151	4.20E-03	1.50E-106	3.89E-10
F24	Mean	4.27E + 03	5.05E + 03	6.72E + 03	7.50E + 03	4.86E + 03	6.26E + 03	**4.20E + 03**	5.37E + 03	4.73E + 03
	STD	1.88E + 02	3.42E + 02	5.00E + 02	5.38E + 02	2.50E + 02	3.08E + 02	**1.04E + 02**	4.69E + 02	6.37E + 02
	p-value	null	1.34E-57	1.23E-108	6.08E-130	3.15E-08	1.09E-122	2.57E-02	9.26E-51	6.89E-67
F25	Mean	**3.97E + 03**	4.85E + 03	9.18E + 03	8.66E + 03	4.58E + 03	1.35E + 04	4.60E + 03	6.24E + 03	9.07E + 03
	STD	**1.48E + 02**	4.28E + 02	1.19E + 03	1.90E + 03	6.57E + 02	1.91E + 03	6.79E + 02	7.19E + 02	8.72E + 03
	p-value	null	1.23E-19	1.86E-47	8.33E-41	5.77E-11	3.15E-53	2.56E-21	9.00E-31	9.32E-44
F26	Mean	2.00E + 04	2.55E + 04	3.16E + 04	2.94E + 04	2.36E + 04	3.67E + 04	**1.71E + 04**	2.74E + 04	1.95E + 04
	STD	2.90E + 03	4.57E + 03	3.84E + 03	4.38E + 03	4.46E + 03	**2.32E + 03**	2.39E + 03	2.97E + 03	3.95E + 03
	p-value	null	3.14E-29	6.11E-100	2.07E-83	**5.71E-01**	7.40E-118	4.56E-41	2.84E-92	7.15E-50
F27	Mean	3.71E + 03	4.35E + 03	5.20E + 03	9.33E + 03	4.07E + 03	6.00E + 03	3.71E + 03	4.55E + 03	**3.20E + 03**
	STD	1.16E + 02	3.10E + 02	6.26E + 02	9.19E + 02	1.91E + 02	7.04E + 02	1.03E + 02	4.36E + 02	**1.27E-04**
	p-value	null	3.93E-64	3.91E-65	1.55E-139	5.25E-58	3.28E-107	8.47E-12	3.67E-86	4.71E-119
F28	Mean	4.18E + 03	5.71E + 03	1.24E + 04	1.13E + 04	5.39E + 03	1.87E + 04	4.54E + 03	8.64E + 03	**3.30E + 03**
	STD	2.08E + 02	7.42E + 02	1.07E + 03	1.39E + 03	7.12E + 02	1.94E + 03	5.59E + 02	9.93E + 02	**2.05E-04**
	p-value	null	1.38E-09	1.16E-77	2.62E-48	6.44E-01	4.38E-76	2.94E-35	1.13E-19	2.24E-137
F29	Mean	**6.95E + 03**	9.12E + 03	1.66E + 04	2.00E + 04	8.37E + 03	2.05E + 04	7.69E + 03	1.05E + 04	1.37E + 04
	STD	6.38E + 02	9.17E + 02	2.43E + 03	9.75E + 03	8.26E + 02	1.18E + 04	**6.24E + 02**	1.14E + 03	6.75E + 03
	p-value	null	8.38E-08	3.07E-83	4.34E-122	5.08E-05	2.07E-78	1.20E-05	1.15E-72	4.49E-82
F30	Mean	**2.45E + 05**	5.19E + 06	2.18E + 09	1.50E + 09	6.99E + 06	1.20E + 10	9.83E + 06	1.20E + 08	5.25E + 07
	STD	**1.36E + 05**	2.82E + 06	1.28E + 09	6.19E + 08	1.10E + 07	5.10E + 09	7.82E + 06	6.69E + 07	1.79E + 08
	p-value	null	4.55E-21	6.04E-65	6.73E-82	2.61E-03	1.41E-117	4.18E-06	1.45E-36	1.64E-22

**Fig. 2:**
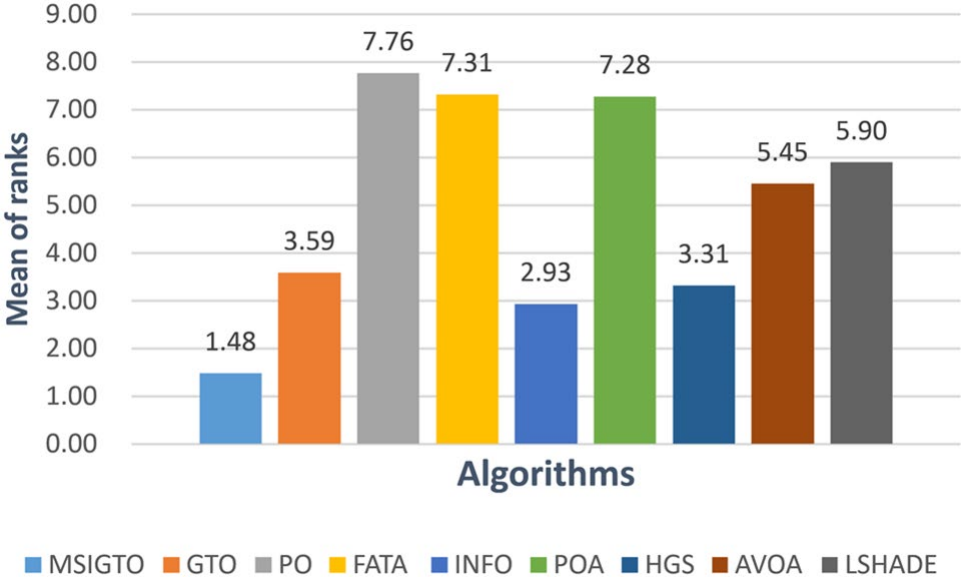
Friedman’s rank test for CEC2017.

To provide a more intuitive comparison of algorithmic performance, the non-parametric Friedman test was employed. The average ranks derived from the Friedman test are illustrated in Fig. 2. As shown, the MSIGTO algorithm achieves an average rank of 1.48, securing the top overall position among all compared algorithms and thereby demonstrating its superior overall performance.

**Fig. 3:**
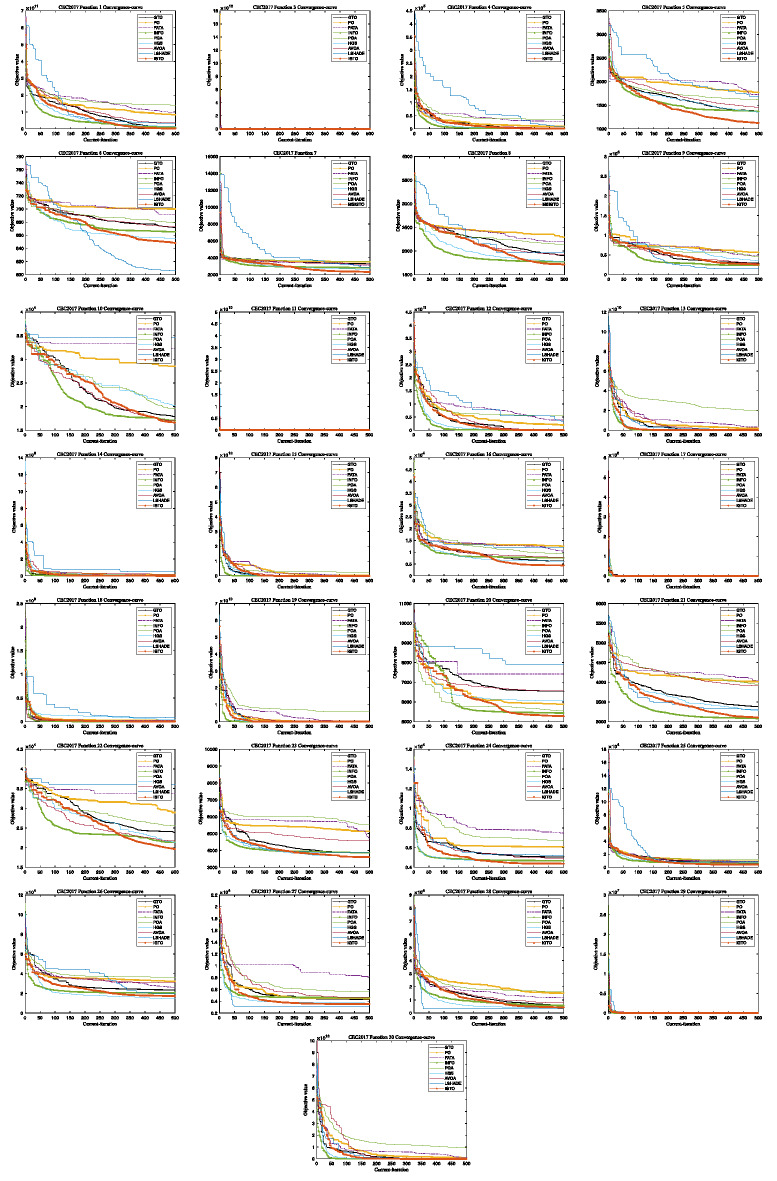
The convergence curves of CEC2017.

Figure 3 depicts the convergence profiles of the algorithms across all iterations for the CEC2017 benchmark functions. It can be observed that MSIGTO converges rapidly and attains the best final solutions on functions F5, F7, F11, F13, F22, F25, and F29. For functions F1, F4, F8, F10, F12, F15–F17, and F19–F21, the algorithm maintains a moderate convergence rate while still reaching optimal outcomes. In contrast, suboptimal results are observed for functions F3, F6, F9, F14, F18, F23, F24, and F26–F28. These results highlight the algorithm’s ability to achieve competitive convergence behavior and reliable solution quality across diverse problem types.

### Results and analysis of the CEC2022 benchmark functions

The experiments are conducted with a maximum of 500 iterations, a population size of 30, and a problem dimensionality of 20. Each algorithm is executed independently 30 times. The CEC2022 benchmark suite consists of 10 test functions, which include one Unimodal Function (F1), four Basic Functions (F2–F5), three Hybrid Functions (F6–F8), and two Composition Functions (F9–F12)^47^. The experimental results, which encompass the mean, standard deviation, and p-values, are presented in Table 4. Based on mean values, MSIGTO achieves the best performance on all functions except F1, F3, F5, F9, and F12. Regarding standard deviation, it performs best on only three functions, indicating that the algorithm’s stability still requires improvement. To assess statistical significance, the Wilcoxon rank-sum test is conducted at a 5% significance level. This non-parametric test evaluates whether the performance disparity between two algorithms is statistically meaningful. A p-value of less than 0.05 is considered indicative of a significant difference. In Table 4, p-values exceeding 0.05 are highlighted in bold, signifying that there is no statistically significant difference. Overall, MSIGTO demonstrates statistically significant improvements over most algorithms, with the exceptions being INFO on F11 and AVOA on F12.

**Table 4:** Performance comparison results of function optimization on CEC2022.

Function	Index	MSIGTO	GTO	PO	FATA	INFO	POA	HGS	AVOA	LSHADE
F1	Mean	2.34E + 03	1.82E + 03	2.13E + 04	1.39E + 04	**1.33E + 03**	1.08E + 04	1.23E + 04	2.76E + 04	6.98E + 04
	STD	1.49E + 03	1.97E + 03	5.20E + 03	5.01E + 03	**1.42E + 03**	3.23E + 03	7.08E + 03	9.36E + 03	2.64E + 04
	p-value	null	2.20E-02	7.43E-97	3.88E-69	7.37E-01	3.41E-24	8.46E-20	6.16E-150	6.35E-183
F2	Mean	**4.24E + 02**	4.65E + 02	5.48E + 02	5.63E + 02	4.56E + 02	6.69E + 02	4.68E + 02	5.10E + 02	4.26E + 02
	STD	**1.73E + 01**	2.33E + 01	4.92E + 01	6.79E + 01	1.90E + 01	1.40E + 02	2.94E + 01	4.41E + 01	2.40E + 01
	p-value	null	5.62E-50	2.31E-99	1.82E-108	2.15E-11	2.21E-95	2.78E-90	9.00E-22	1.08E-07
F3	Mean	6.09E + 02	6.30E + 02	6.57E + 02	6.51E + 02	6.15E + 02	6.50E + 02	6.03E + 02	6.52E + 02	**6.00E + 02**
	STD	8.39E + 00	9.50E + 00	1.04E + 01	8.99E + 00	7.54E + 00	1.00E + 01	2.61E + 00	1.18E + 01	**8.56E-01**
	p-value	null	9.09E-18	1.77E-119	9.91E-91	2.24E-140	4.72E-62	6.34E-104	2.71E-42	6.82E-80
F4	Mean	**8.65E + 02**	8.77E + 02	8.95E + 02	9.07E + 02	8.70E + 02	8.81E + 02	9.14E + 02	8.91E + 02	9.17E + 02
	STD	1.56E + 01	1.60E + 01	1.58E + 01	1.26E + 01	2.05E + 01	**9.90E + 00**	2.62E + 01	2.58E + 01	3.14E + 01
	p-value	null	1.11E-58	4.87E-96	8.02E-69	6.19E-56	5.73E-75	1.15E-26	3.48E-136	1.52E-112
F5	Mean	1.74E + 03	1.99E + 03	2.56E + 03	2.74E + 03	1.52E + 03	2.27E + 03	2.42E + 03	2.42E + 03	**9.83E + 02**
	STD	3.17E + 02	3.50E + 02	3.93E + 02	2.40E + 02	3.20E + 02	2.42E + 02	5.78E + 02	3.20E + 02	**2.09E + 02**
	p-value	null	2.47E-60	4.75E-67	4.77E-41	1.04E-37	7.30E-11	1.00E-35	9.11E-103	3.79E-53
F6	Mean	**5.28E + 03**	5.59E + 03	2.04E + 06	3.26E + 05	6.39E + 03	1.17E + 06	1.47E + 04	8.44E + 03	5.50E + 06
	STD	**4.11E + 03**	5.17E + 03	3.49E + 06	4.44E + 05	5.64E + 03	3.01E + 06	8.36E + 03	6.86E + 03	1.12E + 07
	p-value	null	5.58E-48	1.12E-96	1.14E-99	1.54E-33	1.95E-108	4.40E-28	8.45E-45	2.64E-120
F7	Mean	**2.07E + 03**	2.11E + 03	2.17E + 03	2.16E + 03	2.12E + 03	2.11E + 03	2.10E + 03	2.17E + 03	2.08E + 03
	STD	3.10E + 01	4.68E + 01	3.00E + 01	**1.25E + 01**	5.05E + 01	2.52E + 01	6.63E + 01	5.90E + 01	3.61E + 01
	p-value	null	1.45E-88	3.73E-126	1.26E-109	7.90E-97	4.66E-72	4.05E-47	8.27E-91	8.81E-88
F8	Mean	**2.23E + 03**	2.24E + 03	2.27E + 03	2.27E + 03	2.29E + 03	2.25E + 03	2.27E + 03	2.27E + 03	2.24E + 03
	STD	3.15E + 01	3.57E + 01	5.21E + 01	7.41E + 01	6.22E + 01	4.12E + 01	5.66E + 01	6.03E + 01	**1.20E + 01**
	p-value	null	2.96E-111	8.42E-143	2.19E-110	2.42E-140	9.75E-89	3.36E-140	5.82E-141	2.00E-121
F9	Mean	2.48E + 03	2.48E + 03	2.60E + 03	2.55E + 03	2.48E + 03	2.57E + 03	2.49E + 03	2.50E + 03	**2.47E + 03**
	STD	**3.16E-03**	5.21E-02	5.43E + 01	3.15E + 01	4.17E-03	5.49E + 01	7.80E + 00	1.06E + 01	4.29E-01
	p-value	null	7.14E-03	5.66E-144	1.68E-131	8.25E-27	2.41E-130	2.08E-04	3.60E-76	1.74E-39
F10	Mean	**2.67E + 03**	3.47E + 03	2.84E + 03	4.13E + 03	3.25E + 03	3.61E + 03	2.78E + 03	3.87E + 03	4.10E + 03
	STD	4.31E + 02	1.29E + 03	9.56E + 02	1.10E + 03	5.72E + 02	1.09E + 03	**1.91E + 02**	9.19E + 02	1.43E + 03
	p-value	null	8.46E-164	1.97E-104	1.79E-148	1.91E-155	3.82E-161	8.91E-147	2.99E-159	2.31E-164
F11	Mean	**2.88E + 03**	2.93E + 03	3.98E + 03	6.35E + 03	2.92E + 03	4.77E + 03	2.93E + 03	3.29E + 03	3.11E + 03
	STD	1.25E + 02	1.41E + 02	5.18E + 02	6.53E + 02	**4.22E + 01**	5.72E + 02	1.20E + 02	4.65E + 02	6.03E + 02
	p-value	null	3.11E-39	9.78E-118	1.02E-121	**1.63E-01**	3.03E-86	8.03E-44	4.45E-45	1.08E-56
F12	Mean	2.96E + 03	3.01E + 03	3.03E + 03	3.21E + 03	2.99E + 03	3.06E + 03	2.97E + 03	2.99E + 03	**2.90E + 03**
	STD	1.79E + 01	6.28E + 01	4.21E + 01	1.07E + 02	4.43E + 01	7.64E + 01	2.49E + 01	2.53E + 01	**1.23E-04**
	p-value	null	5.29E-120	3.97E-154	4.54E-155	8.74E-115	1.19E-143	2.23E-122	**6.22E-02**	2.27E-149

**Fig. 4:**
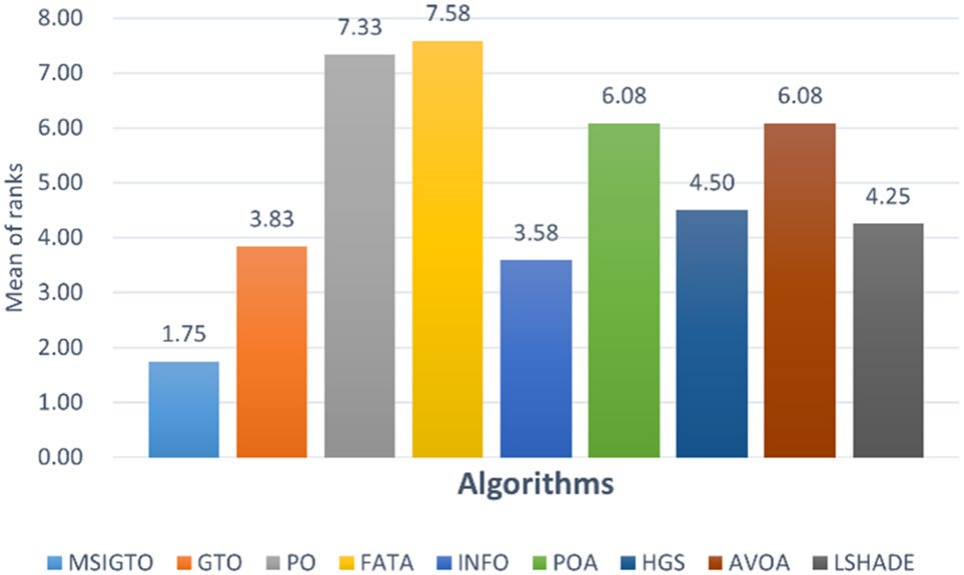
Friedman’s rank test for CEC2022.

To provide a deeper comparison of the algorithms’ performance, the nonparametric Friedman test was applied. Figure 4 displays the average ranks derived from the Friedman test. As illustrated in the figure, the MSIGTO algorithm the MSIGTO algorithm achieves an average rank of 1.75, securing the top overall position among all compared algorithms, emphasizing its outstanding overall performance.

**Fig. 5:**
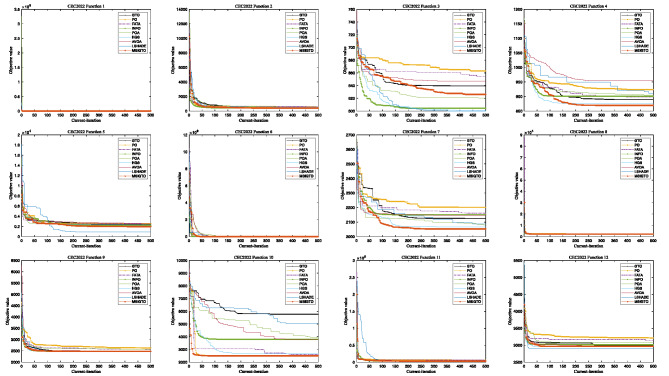
The convergence curves of CEC2022.

To explore valuable regions within the given search space more thoroughly, optimization algorithms typically exhibit large variations in the search individuals during the early stages of iteration. With the increasing number of iterations, these variations progressively converge. The convergence behavior of each algorithm over the full course of iterations on the CEC2022 benchmark is illustrated in Fig. 5. As shown in the figure, functions F7, F8, F10, and F11 exhibit faster convergence and reach the best final values. Functions F2, F4, and F6 show moderate convergence speed while still achieving optimal final solutions. However, functions F1, F3, F5, F9, and F12 fail to attain the optimal value. Overall, the MSIGTO algorithm demonstrates superior performance in both convergence speed and final solution quality.

## Engineering design problems

To assess the real-world applicability of the proposed method, the MSIGTO algorithm is employed to tackle four engineering design problems. These problems are derived from actual industrial processes and are formulated as single-objective optimization models with multiple inequality constraints. The mathematical models involve various types of constraints, including both equality and inequality forms. For comparative analysis, MSIGTO is benchmarked against GTO, PO, FATA, INFO, POA, HGS, and AVOA under identical experimental settings. The population size is fixed at 30, with a maximum of 500 iterations. By using penalty functions to handle constraints, penalty function methods convert constrained optimization problems into unconstrained ones by adding a term that penalizes constraint violations. Feasible solutions remain unchanged, while infeasible solutions incur increasing penalties, guiding the search toward the feasible region. This approach is widely used in engineering due to its simplicity and flexibility.

### Speed reducer design problem

**Fig. 6:**
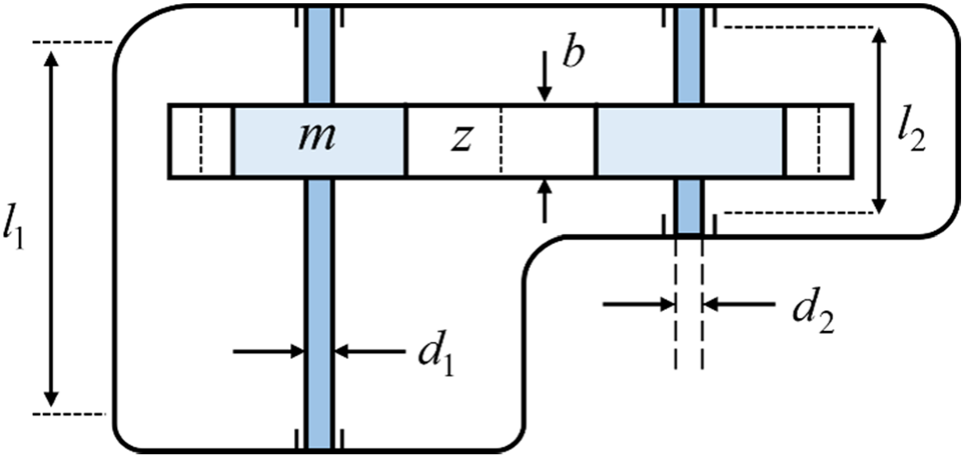
Speed reducer design problem.

This design problem focuses on minimizing the gear reducer’s weight subject to a number of predefined constraints^54^. Figure 6 illustrates that this problem is characterized by seven design variables: gear module (*m*), face width (*b*), number of teeth (*p*), the span between the bearing points on the first shaft (*l₁*), the span between the bearing points on the second shaft (*l₂*), and the diameters of the first and second shafts (*d₁* and *d₂*, respectively). A comparative evaluation of MSIGTO and seven alternative algorithms is summarized in Table 5. The results demonstrate that MSIGTO, along with GTO and HGS, achieves the best optimization performance, with the three yielding closely similar optimal values for the design variables. These outcomes are notably superior to those obtained by the other five algorithms. The formal mathematical representation of the problem is given below:

$$\;\bar {z}=[{z_1},{z_2},{z_3},{z_4},{z_5},{z_6},{z_7}]=[b,m,p,{l_1},{l_2},{d_1},{d_2}]$$

Minimize:

$$\begin{aligned} f(\bar {z}) & =0.7854{z_1}z_{2}^{2}(3.3333z_{3}^{2}+14.9334{z_3} - 43.0934) \\ & \;\;\; - 1.508{z_1}(z_{6}^{2}+z_{7}^{2})\;+7.4777(z_{6}^{3}+z_{7}^{3}) \\ & \;\;+0.7854({z_4}z_{6}^{2}+{z_5}z_{7}^{2}) \\ \end{aligned}$$

Subject to:

$$g_{1} (\bar{z}) = \frac{{27}}{{z_{1} z_{2}^{2} z_{3} }} - 1 \le 0$$

$$g_{2} (\bar{z}) = \frac{{397.5}}{{z_{1} z_{2}^{2} z_{3}^{2} }} - 1 \le 0$$

$$g_{3} (\bar{z}) = \frac{{1.93z_{4}^{3} }}{{z_{2} z_{7}^{4} z_{3} }} - 1 \le 0$$

$$g_{4} (\bar{z}) = \frac{{1.93z_{4}^{3} }}{{z_{2} z_{7}^{4} z_{3} }} - 1 \le 0$$

$$g_{5} (\bar{z}) = \frac{{\left[ {\left( {745(z_{4} /z_{2} z_{3} )} \right)^{2} + 16.9 \times 10^{6} } \right]^{{1/2}} }}{{110z_{6}^{3} }} - 1 \le 0$$

$$g_{6} (\bar{z}) = \frac{{\left[ {\left( {745(z_{5} /z_{2} z_{3} )} \right)^{2} + 157.5 \times 10^{6} } \right]^{{1/2}} }}{{85z_{7}^{3} }} - 1 \le 0$$

$$g_{7} (\bar{z}) = \frac{{z_{2} z_{3} }}{{40}} - 1 \le 0$$

$$g_{8} (\bar{z}) = \frac{{5z_{2} }}{{z_{1} }} - 1 \le 0$$

$$g_{9} (\bar{z}) = \frac{{z_{1} }}{{12z_{2} }} - 1 \le 0$$

$$g_{{10}} (\bar{z}) = \frac{{1.5z_{6} + 1.9}}{{z_{4} }} - 1 \le 0$$

$$g_{{11}} (\bar{z}) = \frac{{1.1z_{7} + 1.9}}{{z_{5} }} - 1 \le 0$$

Where:

$$\begin{gathered} 2.6 \le z_{1} \le 3.6,\;0.7 \le z_{2} \le 0.8,\;17 \le z_{3} \le 28,\;7.3 \le z_{4} \le 8.3, \hfill \\ 7.3 \le z_{5} \le 8.3,\;2.9 \le z_{6} \le 3.9,\;5.0 \le z_{7} \le 5.5 \hfill \\ \end{gathered}$$

**Table 5:** Performance comparison for speed reducer design Problem.

Algorithms	b	m	*p*	l1	l2	d1	d2	Optimum cost
MSIGTO	3.499999759	0.7	17	7.3	7.71532	3.350541	5.286654	**2994.554140128**
GTO	3.499999759	0.7	17	7.3	7.71532	3.350541	5.286654	**2994.554140128**
PO	3.50730322	0.7	17	8.177301413	8.139766	3.354118	5.287025	3015.640910385
FATA	3.505310316	0.7	17	7.3	8.152516	3.354906	5.291733	3010.597841186
INFO	3.499999759	0.7	17	7.3	7.71532	3.350541	5.286654	**2994.554140128**
POA	3.502320735	0.7	17	7.301948305	7.716625	3.373573	5.286745	3001.479536479
HGS	3.599999962	0.7	17	7.3	7.71532	3.350541	5.286654	3033.831302748
AVOA	3.500038166	0.7	17.00000885	8.297455147	7.748413	3.352569	5.286682	3004.636791351

Optimal values are in [bold].

### Gear train design problem

**Fig. 7:**
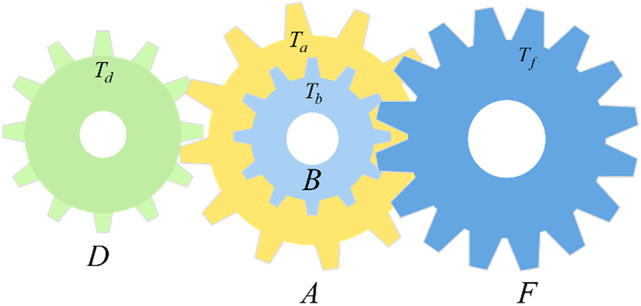
Gear train design problem.

The gear train design problem aims to minimize the cost of achieving a specific transmission ratio in a compound gear train^55^. The gear transmission ratio describes the relationship between the angular velocities of the output and input shafts. As depicted in Fig. 7, the compound gear system includes two gear pairs, B–F and D–A, to meet the desired total transmission ratio. Four integer variables are considered in this problem, where *T*_*a*_, *T*_*b*_, *T*_*d*_, and *T*_*f*_ denote the number of teeth assigned to individual gears. As detailed in Table 6, MSIGTO is evaluated alongside seven other optimization algorithms. Notably, MSIGTO, POA, and HGS successfully obtain the optimal result, significantly outperforming their counterparts. The following equations define the mathematical model:

$$Variablex=[{T_a},{T_b},{T_d},{T_f}]$$

$$\hbox{min} f(x)={(\frac{1}{{6.931}} - \frac{{{T_b} \cdot {T_d}}}{{{T_a} \cdot {T_f}}})^2}$$

Subject to:

$${x_i} \in {N_+},i=1,2,3,4$$

Variable range:

$$0.01 \leqslant {x_i} \leqslant 60,i=1,2,3,4$$

**Table 6:** Performance comparison for gear train design problem.

Algorithms	T_a_	T_b_	T_d_	T_f_	Optimum cost
MSIGTO	50.91175012	13.22453865	29.68387575	53.46992555	**2.30782E-11**
GTO	58.97454351	13.07575395	38.38102085	58.44949355	6.51226E-09
PO	54.42123808	37.41289675	12	57.17199173	8.88761E-10
FATA	53.68758544	12	37.17664804	57.48281511	8.88761E-10
INFO	58.43542179	30.08805929	12.41256628	43.2350941	4.5033E-09
POA	53.28124589	25.5244994	14.62013288	51.07291044	**2.30782E-11**
HGS	34.46042878	19.76797068	13.11047771	52.88360276	**2.30782E-11**
AVOA	48.68567701	32.73498475	12	56.42773848	1.26338E-09

Optimal values are in [bold].

### Multiple disk clutch brake design problem

**Fig. 8:**
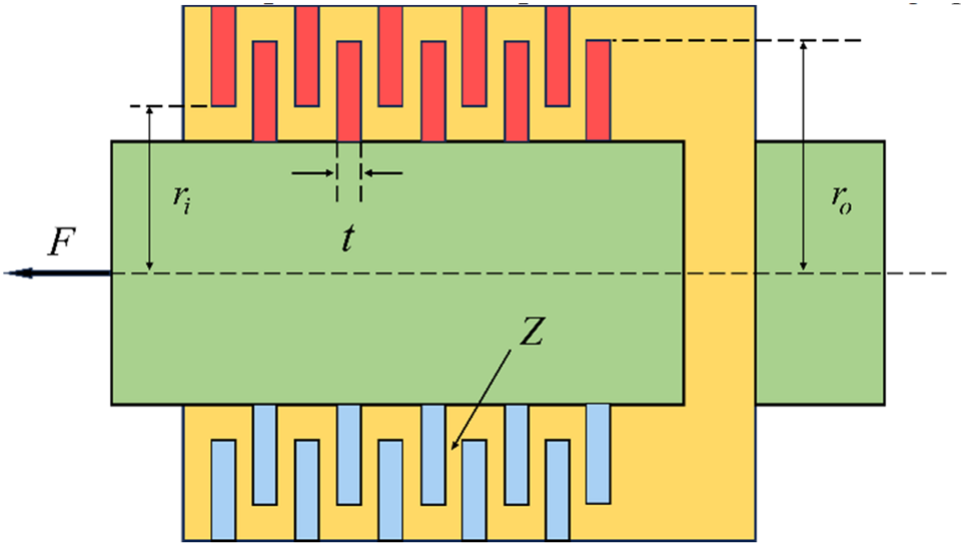
Multiple disk clutch brake design problem.

The main goal in this optimization task is the reduction of the mass of a multi-disc clutch brake^56^. Five integer decision variables are considered in this problem: inner radius (*r*_*i*_), outer radius (*r*_*o*_), disc thickness (*t*), force applied (*F*), and the total number of friction surfaces (*Z*). Figure 8 demonstrates the structural design variables of the problem. In Table 7, the MSIGTO algorithm is benchmarked against seven metaheuristic approaches. According to the results, MSIGTO, GTO, INFO, POA, and HGS all reach the optimal solution, yielding comparable values for the decision variables. The following equations define the mathematical model:

Minimize:

$$f(\overline{x}) = \pi (x_{2}^{2} - x_{1}^{2} )x_{3} (x_{5} + 1)_{\rho }$$

Subject to:

$$g_{1} (\overline{x}) = - p_{max} + p_{rz} \le 0$$

$$g_{2} (\overline{x}) = p_{rz} \nu_{sr} - \nu_{sr,maxPmax} \le 0$$

$$g_{3} (\overline{x}) = {\Delta }R + x_{1} - x_{2} \le 0$$

$$g_{4} (\overline{x}) = - L_{max} + (x_{5} + 1)(x_{3} + \delta ) \le 0$$

$$g_{5} (\overline{x}) = sM_{s} - M_{h} \le 0$$

$$g_{6} (\overline{x}) = T \ge 0$$

$$g_{7} (\overline{x}) = - \nu_{sr,max} + \nu_{sr} \le 0$$

$$g_{8} (\overline{x}) = T - T_{max} \le 0$$

where:

$$M_{h} = \frac{2}{3}\mu x_{4} x_{5} \frac{{x_{2}^{3} - x_{1}^{3} }}{{x_{2}^{2} - x_{1}^{2} }}N.mm$$

$$\omega = \frac{\pi n}{{30}}rad/s$$

$$A = \pi (x_{2}^{2} - x_{1}^{2} )mm^{2}$$

$$p_{rz} = \frac{{x_{4} }}{A}N/mm^{2}$$

$$\nu_{sr} = \frac{{\pi R_{sr} n}}{30}mm/s$$

$$R_{sr} = \frac{2}{3}\frac{{x_{2}^{3} - x_{1}^{3} }}{{x_{2}^{2} x_{1}^{2} }}mm$$

$$T = \frac{{I_{z} \omega }}{{M_{h} + M_{f} }}$$

$$\begin{gathered} {\Delta }R = 20mm,L_{max} = 30mm,\mu = 0.6, \hfill \\ V_{sr,max} = 10m/s,\delta = 0.5mm,s = 1.5 \hfill \\ \end{gathered}$$

$$\begin{gathered} T_{max} = 15s,n = 250rpm,T_{z} = 55Kg.m^{2} , \hfill \\ M_{s} = 40Nm,\;M_{f} = 2Nm,\;p_{max} = 1 \hfill \\ \end{gathered}$$

Variable range:

$$\begin{gathered} 60 \le x_{1} \le 80,\;90 \le x_{2} \le 110,\;1 \le x_{3} \le 3, \hfill \\ 0 \le x_{4} \le 1000,\;2 \le x_{5} \le 9 \hfill \\ \end{gathered}$$

**Table 7:** Performance comparison for multiple disk clutch brake design problem.

Algorithms	ri	ro	t	F	Z	Optimum cost
MSIGTO	70	90	1	1000	2	**0.235242458**
GTO	70	90	1	23.65182755	2	**0.235242458**
PO	69.9995866	90	1	263.3123978	2	0.235246713
FATA	69.99754404	90	1	425.6541455	2	0.235267734
INFO	70	90	1	991.5261322	2	**0.235242458**
POA	70	90	1	1000	2	**0.235242458**
HGS	70	90	1	317.2758299	2	**0.235242458**
AVOA	69.99738773	90	1	619.1675363	2	0.235269343

Optimal values are in [bold].

### SOPWM for 3-level inverters

**Fig. 9:**
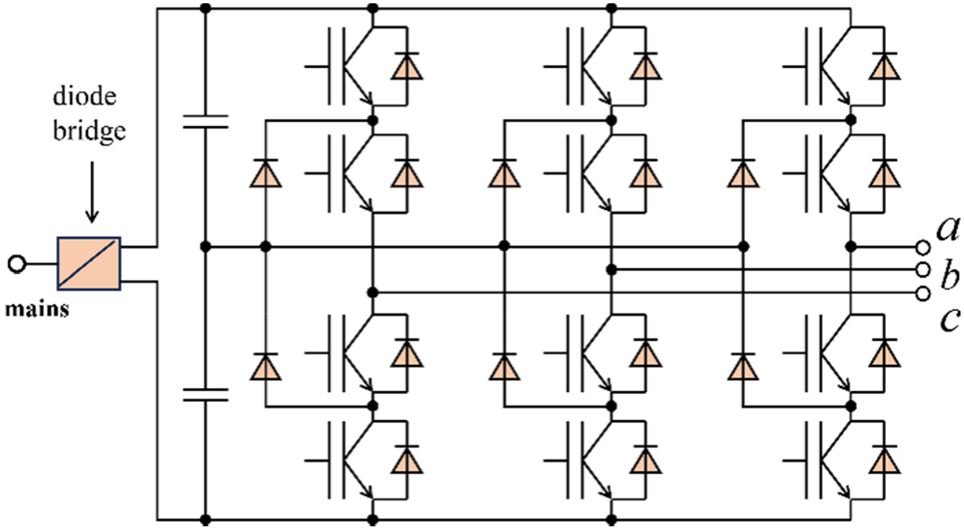
Three-level NPC inverter topology.

The control of medium voltage (MV) drives has recently seen the adoption of Synchronous Optimal Pulse Width Modulation (SOPWM) as a promising approach^57^. The objective of this design problem is to compute switching angles within a fundamental cycle to minimize current harmonic distortion. In the experiment, *f*_*s, max*_ represents the maximum switching frequency, set at 200, and *f*_*m*_ represents the fundamental frequency, set at 50. The switching angles are the input values for the problem. A three-level inverter is illustrated in Fig. 9. Table 8 indicates that MSIGTO surpasses the competing algorithms, highlighting its strong performance in handling constrained optimization problems. The following equations define the mathematical model:

Minimize:

$$f = \frac{{\sqrt {\mathop \sum \limits_{k} (k^{{ - 4}} )\left( {\mathop \sum \limits_{{i = 1}}^{N} s(i)cos(k\alpha _{i} )} \right)^{2} } }}{{\sqrt {\mathop \sum \limits_{k} k^{{ - 4}} } }}$$

Where:

$$k = 5,7,11,13 \ldots ..97,N = \left[ {\frac{{f_{s,max} }}{f.m}} \right],\;s(i) = ( - 1)^{i + 1}$$

Subject to:

$$g_{i} = \alpha_{i + 1} - \alpha_{i} - 10^{ - 5} > 0,i = 1,2, \ldots N - 1$$

$$h_{1} = m - \mathop \sum \limits_{{i = 1}}^{N} s(i)cos(\alpha _{i} ) = 0$$

Variable range:

$$0 < \alpha_{i} < \frac{\pi }{2},i = 1,2,...N$$

**Table 8:** Performance comparison for SOPWM for 3-level inverters.

Algorithms	α1	α2	α3	α4	Optimum cost
MSIGTO	0.512284107	1.549129340	1.570758218	1.570796327	**1.129791881**
GTO	0.041972335	0.047879215	0.059703813	1.421761692	1.788054155
PO	0.473890826	1.478152067	1.516695081	1.569432855	1.154999372
FATA	0.404643488	1.296452474	1.301520301	1.506409687	4.747203740
INFO	0.010770864	0.628421125	0.851288373	1.570796327	1.453183545
POA	0.390940191	0.565556259	0.574293255	1.500902988	1.458714669
HGS	0	0.000056702	0.554811036	1.570796327	1.675648620
AVOA	0.554807795	1.570793457	1.570795016	1.570796327	2.580323059

Optimal values are in [bold].

## Discussion

This study comprehensively evaluates the MSIGTO algorithm across two benchmark suites—CEC2017 (100D) and CEC2022 (20D)—as well as four practical engineering optimization problems. The results demonstrate that MSIGTO generally performs well, particularly on hybrid and composition functions with complex landscapes where global search and local exploitation must be balanced. In the high-dimensional CEC2017 tests, MSIGTO secures the top rank and achieves the best mean results on 20 out of 29 functions, indicating that the integrated strategies effectively maintain diversity and escape local minima. By contrast, its advantage diminishes on several simple unimodal functions in CEC2022 (e.g., F1, F3, F5), partly due to excessive exploration that slows convergence. This phenomenon aligns with the No Free Lunch (NFL) theorem, which asserts that no algorithm consistently outperforms others across all problem types. Convergence analysis also confirms that MSIGTO descends rapidly during early iterations on most problems and achieves competitive final precision. In the engineering tasks, the algorithm consistently reaches optimal or near-optimal designs and adapts well to mixed discrete–continuous decision spaces.

Despite these strengths, two limitations are observed. First, the algorithm’s stability and robustness remain improvable, as performance variance persists on low-dimensional problems with sharp optima. Second, this work focuses solely on single-objective optimization; its effectiveness on multi-objective formulations has yet to be validated. Overall, the combination of Latin Hypercube Sampling, Lévy Flight, and the Cauchy operator yields a favorable exploration–exploitation balance, enabling MSIGTO to tackle high-dimensional and constrained problems effectively. Future research may incorporate adaptive parameter control to enhance stability and extend its applicability to multi-objective and dynamic optimization scenarios.

## Conclusion

This paper introduces MSIGTO, an enhanced variant of the GTO algorithm. It integrates Latin Hypercube Sampling, Cauchy Inverse Cumulative Distribution, and Lévy Flight to boost both the initial population diversity and the global optimization efficiency of the original method. The performance of MSIGTO is assessed with respect to its exploration and exploitation abilities, robustness against local optima, and solution stability through both benchmark test functions and practical engineering optimization tasks. The results are compared with those of PO, FATA, INFO, POA, HGS, AVOA, LSHADE, and the original GTO algorithm. The IEEE CEC2017 benchmark and IEEE CEC2022 benchmark are utilized to provide further evidence of MSIGTO’s effectiveness in addressing complex and high-difficulty composition functions. The test results show that MSIGTO performs well across most functions, yielding highly competitive results. Furthermore, to validate the practical applicability of MSIGTO, four constrained engineering design problems are selected for evaluation: the Speed Reducer design problem, Gear Train design problem, Multiple Disk Clutch Brake design problem, and SOPWM for 3-level Inverters. The results demonstrate that MSIGTO exhibits excellent engineering application capabilities and strong problem-solving abilities in unknown spaces.

In future work, several promising directions can be pursued to further enhance the MSIGTO framework. From an algorithmic perspective, incorporating memory-saving mechanisms or adaptive parameter decay strategies could reduce computational overhead while improving convergence stability. Moreover, the current study is limited to single-objective optimization; extending MSIGTO to handle multi-objective or many-objective problems would enable it to address more complex real-world scenarios with conflicting objectives. Another important avenue lies in improving robustness under noisy or dynamic environments, which is essential for real-time engineering applications. Beyond benchmark testing, future investigations may also consider applying the algorithm to emerging domains such as dynamic task scheduling, supply chain network optimization, large-scale Internet of Things (IoT) systems, and intelligent medical diagnostics, thereby evaluating its generalization ability and practical value across diverse application contexts. Additionally, hybridization with other metaheuristic or machine learning methods, such as surrogate modeling or reinforcement learning, may further enhance its exploration–exploitation balance and adaptability to high-dimensional spaces.

## Data Availability

All data generated or analyzed during this study are included in this published article.
